# Male Infertility Knowledgebase: decoding the genetic and disease landscape

**DOI:** 10.1093/database/baab049

**Published:** 2021-08-07

**Authors:** Shaini Joseph, Smita D Mahale

**Affiliations:** Genetic Research Center, ICMR-National Institute for Research in Reproductive Health, J.M. Street, Parel, Mumbai 400012, India; Emeritus Scientist, ICMR-National Institute for Research in Reproductive Health, J.M. Street, Parel, Mumbai 400012, India

## Abstract

Male infertility is a multifactorial condition that contributes to around one-third of cases of infertility worldwide. Several chromosomal aberrations, single-gene and polygenic associations with male factor defects have been reported. These defects manifest as sperm number or sperm quality defects leading to infertility. However, in almost 40% of cases, the genetic etiology of male infertility remains unexplained. Understanding the causal genetic factors is crucial for effective patient management and counseling. Integrating the vast amount of available omics data on male infertility is a first step towards understanding, delineating and prioritizing genes associated with the different male reproductive disorders. The Male Infertility Knowledgebase (MIK) is a manually curated repository developed to boost research on the elusive genetic etiology of male infertility. It integrates information on ∼17 000 genes, their associated pathways, gene ontology, diseases and gene and sequence-based analysis tools. In addition, it also incorporates information on reported chromosomal aberrations and syndromic associations with male infertility. Disease enrichment of genes in MIK indicate a shared genetic etiology between cancer, male and female infertility disorders. While the genes involved in cancer pathways were found to be common causal factors for sperm number and sperm quality defects, the interleukin pathways were found to be shared and enriched between male factor defects and non-reproductive conditions like cardiovascular diseases, metabolic diseases, etc. Disease information in MIK can be explored further to identify high-risk conditions associated with male infertility and delineate shared genetic etiology. Utility of the knowledgebase in predicting novel genes is illustrated by identification of 149 novel candidates for cryptorchidism using gene prioritization and network analysis. MIK will serve as a platform for review of genetic information on male infertility, identification pleiotropic genes, prediction of novel candidate genes for the different male infertility diseases and for portending future high-risk diseases associated with male infertility.

**Database URL**: http://mik.bicnirrh.res.in/

## Introduction

Male factor defects contribute to ∼30% cases with infertility across the globe (1). Several factors ranging from infections, hormonal dysfunction to structural defects (2, 3) could cause these defects leading to male infertility. Although the clinical presentation of subjects with male infertility is heterogeneous and complex, the causal factor in about 15% of cases is due to either chromosomal aberrations or single-gene alterations (4–6). The genes associated with male infertility include genes involved in various processes like spermatogenesis (e.g. *USP9Y, DBY, RBMY, TEX11*, *DAZ*) (7–9), development of the male reproductive system (e.g. *AR, FSHR, CFTR, ADGR2*) (9, 10), steroid hormone signaling (e.g. *SHBG*) (11), etc. Defects in genes associated with male infertility manifest as either qualitative or quantitative sperm defects leading to infertility (12). Presently existing cytogenetic techniques, microarray and sequencing of specific male infertility gene panels have identified several genetic causes of male infertility (13). However, the genetic etiology in 40% of male infertility cases remains unidentified (12, 14).

The current lacunae of idiopathic or unexplained male infertility is due to paucity in studies on polygenic causes of male infertility. The lack of identification of the complete repertoire of genes associated with male infertility, the disparity in existing studies across populations and the dearth of studies on functional effects of identified mutations and epimutations in genes associated with male infertility make it difficult to understand the genetic etiology of male infertility (13).

The plausibility of employing high-throughput technologies like next-generation sequencing and genome-wide association studies have led to the identification of several novel candidate genes with a probable role in male infertility (12). Currently, over thousand genes have been reported to play a role in reproductive processes crucial for male fertility. An aberration in one of these genes can affect the expression and activity of its interacting partners and downstream genes, thus hampering hormonal regulation, spermiogenesis, spermatogenesis or lead to structural defects (15).

Understanding the genetic cause of male infertility is clinically important for better prognosis, treatment and to assess the risk of transmission of genetic defects through natural or assisted reproductive techniques (16, 17). It is also important to understand and to predict the risk of other diseases in patients with male infertility and decide appropriate treatment modalities (16). Recently, several studies have focused on understanding the high-risk disease conditions associated with male infertility (18, 19). In some cases, the manifestation of infertility indicated the risk of a future health concern (20). Males suffering from infertility have been known to have a higher risk of testicular cancer (21, 22), prostate cancer (21), cardiovascular diseases (23, 24) and metabolic syndrome (19, 25, 26).

Common genetic variations resulting in infertility and other high-risk disease conditions need to be investigated further so as to benefit clinicians in appropriate patient management (27, 28). The advent of omics data has paved the way for employing computational methods to integrate, analyze and infer data with high confidence (15). A gene-based database with integrated information is a prerequisite for these computational studies.

Here, we present the Male Infertility Knowledgebase (MIK), a resource with compiled information on all reported genetic causes of male infertility mined from the PubMed database. The knowledgebase also includes information on literature-supporting congenital causes of male infertility and data generated by high-throughput technologies. Genes in the knowledgebase have been annotated with information on the known genetic aberrations related to male infertility, functional and pathway information. Disease conditions that share the genetic etiology are also included in this database. The information present in this resource will boost research in the area of genetics of male infertility and will help in the better understanding of the complex genetic etiology of male infertility.

## Methods

### Data collection

MIK was developed to hold information on genes reported in the literature to be associated with defects leading to male infertility. The PubMed (29) database was iteratively searched by combining gene synonyms with infertility keywords: ‘infertility’, ‘fertility’, ‘male infertility’, ‘azoospermia’, ‘aspermia’ ‘oligozoospermia’, ‘teratozoospermia’, ‘necrozoospermia’, ‘asthenozoospermia’, ‘cryptorchidism’, ‘hypospadias’, ‘sperm defects’ and ‘varicocele’. The literature references retrieved were subjected to manual curation to mine for information on population under study, ethnicity, SNPs reported, associated comorbid conditions, other genes studied, etc. The genes with an associated role in male infertility were divided into two datasets i.e. the validated dataset and the predicted dataset. While the validated dataset includes genes that were retrieved from candidate gene and/or functionally validated studies, the predicted dataset includes genes retrieved from high-throughput studies reported in the paper or analyzed using the GEO2R tool (30).

A huge proportion of male fertility defects are due to chromosomal aberrations or syndromes. Literature references on syndromes citing male factor defects as a phenotypic manifestation are included as a separate section under syndromes. Chromosomal aberrations that are reported as causal factors for male infertility are categorized separately under chromosomal defect section.

The genes in the database are annotated with information on known SNPs from the SNP database (29), protein details from the UniProt database (31), gene ontology information from the AMIGO2 database (32, 33), pathway information extracted from the KEGG pathways database (34) and associated diseases collated from the Genetic Association Database (GAD) (35), KEGG disease database and in the Online Mendelian Inheritance in Man database (36). The compiled list of gene–disease associations was further manually curated to remove redundant entries. Systemic diseases like all types of cancers and immune system diseases are grouped into broad categories as cancer and immune system disorders, respectively. The other diseases that affect a target organ or tissue are classified based on MeSH categories (29).

### Database architecture

The knowledgebase is developed using MySQL Server 5.1.33, and the user interfaces are created using PHP 5.2.9, HT;ML and JavaScript. Apache HTTP Server 2.2.11 is used as the server for the knowledgebase.

### Database design

A user-friendly database interface was designed for MIK. The objective and the utility of the database have been explained on the Home Page. Information present in the database is segregated and displayed on the Database Statistics Page. A help page explaining the different search options and tools of the database has been included.

Search and browse pages are designed to facilitate efficient searches and download of data. The Validated and Predicted (Highthroughput) dataset can be retrieved using the advanced search option (Gene and Literature Reference link). In addition to simple and advanced search options, four special search options included in MIK are described below:

Search Syndromes: This option can be used to retrieve information on syndromes associated with male infertility reported in the literature.

Search Chromosomal defects: This option can be used to retrieve information on chromosomal aberrations associated with male infertility reported in the literature.

Search Infertility phenotypes: The data in the validated dataset of MIK have been broadly classified into 21 different groups for efficient search and analysis.

Search Highthroughput dataset: The predicted dataset with genes indentified from highthrouput experiments (complete and individual male infertility phenotypes) were prioritized using the validated dataset (complete and individual male infertility phenotypes) respectively using the ToppGeneSuite (37). The prioritized rank/*P*-value based on functional information and protein–protein interaction network can be accessed using this link.

Additionally, the third option of prioritization based on a number of evidence/references in MIK (predicted dataset) has also been included.

The tools included in the knowledgebase are segregated into three sections described below:

Tools: This section provides the CDART (38) and the Motif Scan (39) hyperlink to identify functionally important regions in the user-selected gene. End users can also check for orthologues of gene of interest and identify effects of the different SNPs present in the knowledgebase using the different programs in g:Profiler (40). The interacting partners of the user-selected genes of interest can be identified from the STRING database interface (41).

Blast: Homologous nucleotide or protein sequences can be retrieved using the BLAST link (42).

Analysis: The common diseases, pathways and gene ontology’s shared amid the user-selected genes can be studied using this feature of the database. g:Profiler and PANTHER database (43) links are also incorporated in the knowledgebase.

### Data analysis

#### MIK data analysis

The unique male infertility phenotypes for each gene were retrieved from MIK validated dataset. The number of genes and their corresponding number of causal male infertility phenotypes were calculated to identify gene overlap between the different male infertility phenotypes. To plot a phenogram and to enable the identification of chromosomal regions enriched in genes associated with male infertility, the information on genes and their corresponding chromosomal location and male infertility phenotype were retrieved. The male infertility phenotypes were broadly grouped into 13 categories that can be accessed through the Search Infertility Phenotypes link in the knowledgebase.

Pleiotropic genes associated with five or more male infertility phenotypes retrieved for network analysis and functional enrichment. These data were analyzed to identify common enriched biological processes affecting multiple male infertility phenotypes. Further, the genes with more than 15 references were retrieved and the strength of their association with the different male infertility phenotypes was measured based on the number of references for each gene–disease association in MIK.

To identify enriched biological process, pathways and diseases for the different male infertility phenotypes independently, the data in MIK validated dataset were segregated into 14 groups. The ToppGene Server was used for gene enrichment. The genes associated with sperm number defects, sperm morphological defects and sperm motility defects were retrieved and were analyzed further to identify genetic overlap and enriched pathways and diseases common to these three common sperm defects.

The future high-risk disease conditions based on genetic overlap were identified by analyzing the genes shared between the different male infertility phenotype and disease (other than diseases of the reproductive system) information present in MIK.

#### Case study

Cytoscape v3.7 (44) was used for network analysis of genes in MIK. The ClueGo plugin (45) in Cytoscape and ToppGene server was used to understand the enriched gene ontology, pathway and diseases among the genes in MIK.

The male infertility diseases in MIK were broadly classified into 14 groups. Cryptorchidism, a multifactorial male infertility disease, was selected to demonstrate the use of MIK in the identification of novel gene–disease associations. Unclear genetic etiology, the presence of gene expression datasets in MIK and several reported candidate genes in MIK made cryptorchidism an appropriate choice for the case study.

To identify novel candidate genes for cryptorchidism, the ToppGene gene prioritization server was used. Human protein-coding genes were retrieved from NCBI gene database. These genes were prioritized using the validated dataset of genes in MIK. Simultaneously, the GEO2R tool in NCBI GEO database was used to analyze expression datasets GSE16191 and GSE25518 (46). The genes with log fold change above 1.5 were shortlisted and pooled with the genes obtained from gene prioritization. Genes with interactions with cryptorchidism-associated genes were obtained from STRING. The MCODE plugin (47) was used to retrieve highly connected clusters in the network and to identify novel candidates from the STRING interaction network. This list was appended to the list of predicted candidate genes. The genes obtained from the predicted dataset of MIK were also pooled to the list of proposed candidate genes. Duplicate entries were removed. The genes were scored based on their rank in the gene prioritization result, their expression in the above-mentioned gene expression datasets, their association with known cryptorchidism-associated genes and their presence in the predicted dataset of MIK. The genes that were identified in more than two of the above approaches were shortlisted as novel predicted candidates for cryptorchidism (Supplementary Figure S1).

## Results and discussion

**Figure 1.**
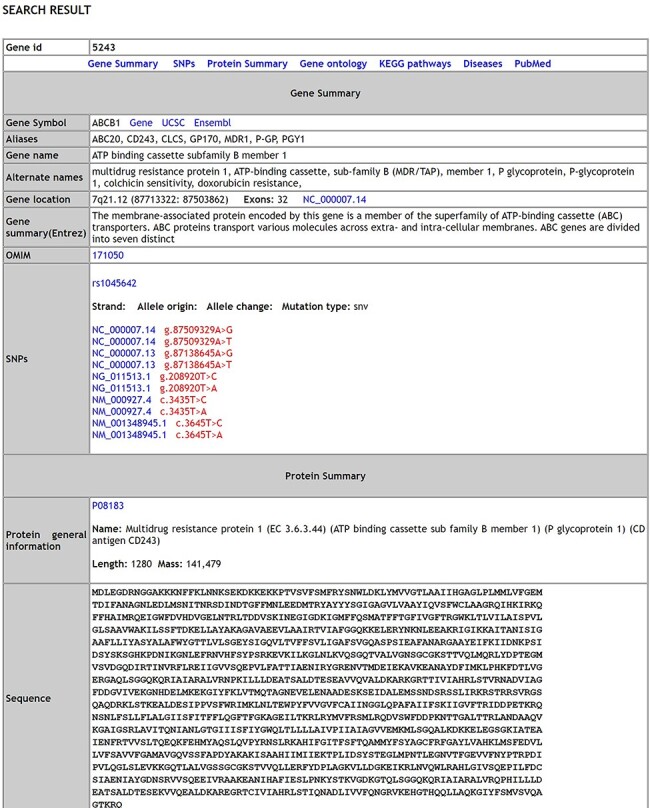
The Search results page of MIK.

A comprehensive list of 17 754 genes [validated dataset: 1564 genes; predicted (Highthroughput) dataset: 16 190 genes] with 738 SNPs, 338 distinct pathways, 8152 distinct disease conditions and 17 870 gene ontology terms describing the genes is integrated in MIK. The database has over 292 syndromes that have reported male infertility as a phenotypic manifestation and over 650 chromosomal aberration causing male factor defects leading to infertility. A snapshot of the database schema (Supplementary Figure S2) and the database results page is shown in Figure 1.

### Identification of gene overlap between the different male infertility phenotypes

**Figure 2.**
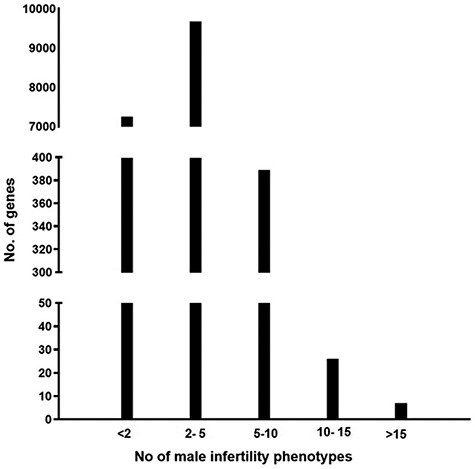
A graph representing the number of genes associated with multiple male reproductive disorders leading to infertility.

The information present in the knowledgebase was analyzed to understand if there is a genetic overlap between the different male reproductive disorders leading to infertility. It was observed that around 58% of genes contribute to more than one aberrant reproductive phenotype leading to infertility (Figure 2)

### Identifying the chromosomal regions enriched in genes associated with male reproductive disorders

**Figure 3.**
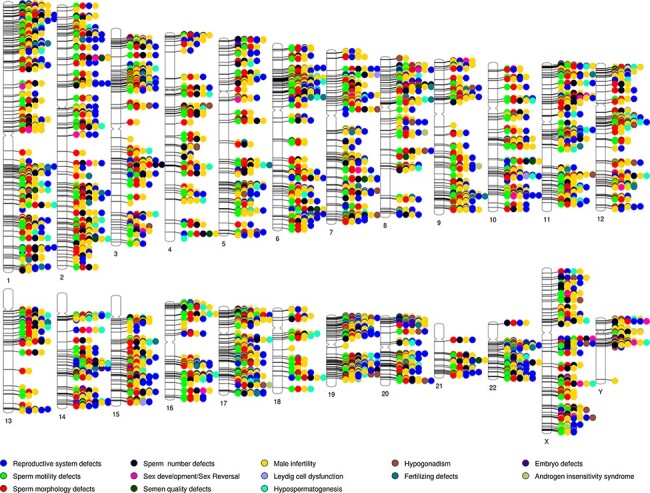
Phenogram showing chromosomal location of genes associated with the different male reproductive diseases in MIK.

A phenogram of the validated dataset in MIK was plotted to identify enriched chromosomal loci associated with male infertility and to visualize their pleiotropic nature (48) (Figure 3). To enable a good resolution, only genes from the validated datasets were used and diseases were broadly grouped into 13 categories. All structural defects were included under reproductive system defects.

While the genes associated with male infertility were spread across all chromosomes, it was observed that in addition to the chromosomes X and Y, chromosomes 6, 17, 19, 20 and 22 have ∼10% genes associated with male infertility. The 6p21.3 and Yq11.223 cytobands were found to be enriched among the validated genes. It is also observed that almost all the loci are shared loci for two or more reproductive disorders. MIK would prove to be a useful resource to retrieve information on the gene affecting multivariate phenotypes of male infertility.

### Analyzing genes that are shared between the different male infertility reproductive phenotypes

**Figure 4.**
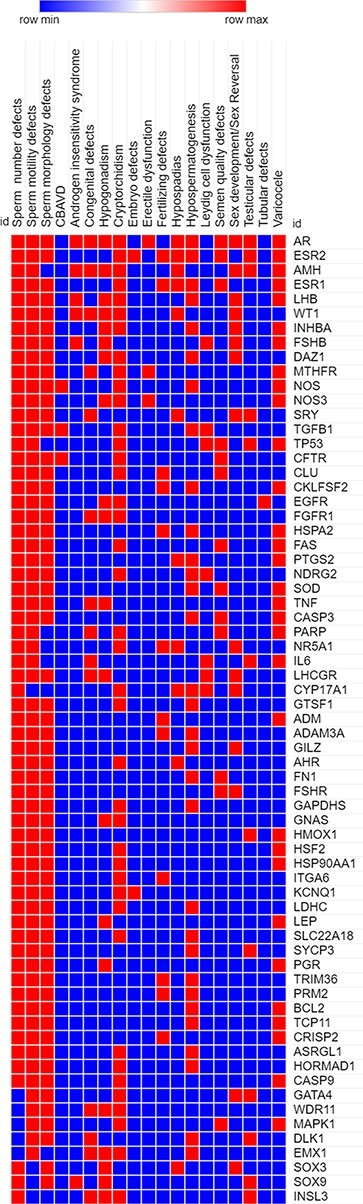
A heatmap representing the association (in red) and no association (in blue) of genes with the different reproductive disorders indexed in MIK.

Highly pleiotropic genes associated with five (∼25%) or more of male infertility diseases in MIK were shortlisted. These genes and their associated diseases are represented in the heatmap (Figure 4).

**Figure 5.**
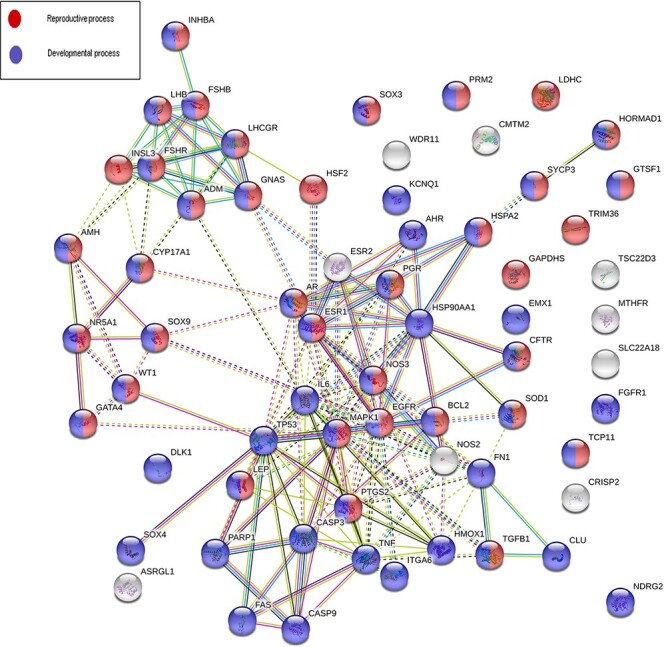
An interaction network on gene products associated with five or more reproductive conditions leading to male infertility.

Sixty-eight genes in MIK were found to be associated with five or more male infertility diseases in MIK. The interaction network of these genes was analyzed to identify biological processes crucial for male fertility. Protein–protein interaction network for these 68 genes was retrieved and clustered using k-means clustering option in the STRING database. The network was analyzed to identify the enriched biological processes among these genes. Eighty-seven percent of these genes were found to be involved in processes crucial for reproduction and development (Figure 5). The mechanism by which these genes affect the different male infertility diseases needs to be further studied.

### Measuring the strength of gene–disease association based on the percentage of citations present in MIK

**Figure 6.**
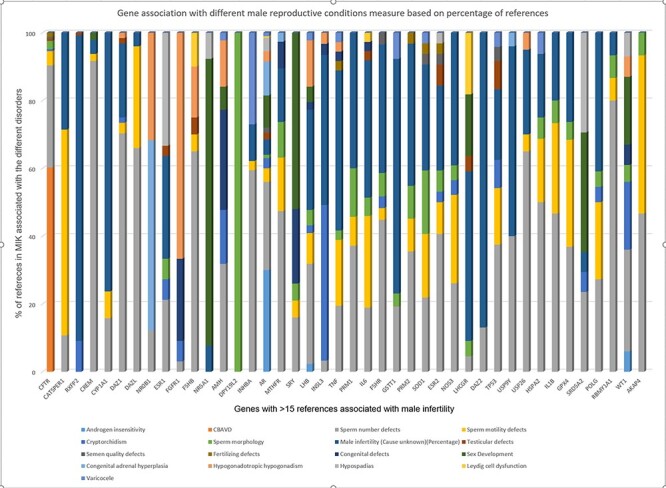
Strength of gene association with different male reproductive conditions based on percentage of references present in MIK.

Next, we tried to understand the strength of association of the pleiotropic genes with each of its associated phenotypes based on the published literature. The strength of gene–disease association was measured based on the percentage of literature references. The cutoff of 15 references was considered suitable to measure gene–disease association based on the gene-association data present in MIK (Figure 1). The genes with more than 15 references in MIK and their corresponding disease association percentage was plotted and is shown in Figure 6.

While the majority of the genes in Figure 6 showed a small percentage of references citing their association with all sperm defects (including sperm number, sperm morphology and sperm motility defects), it was observed that some of the genes show a stronger disease association with one of the phenotypes. *DAZ1, DAZL, FSHB, GSTM1, HSPA2, FAS, INHBA, RBMY1A1* genes were most frequently cited for sperm number defects, *DPY19L*2 was found to be reported only for sperm morphological defects, while *CATSPER1* was found mostly associated with sperm motility.

The single major gene responsible for agenesis of vas deferens based on data in MIK was *CFTR. NR5A1* and *SRY* were mostly reported for sex developmental disorders, NR0B1 for congenital adrenal hyperplasia and *FGFR1* for hypogonadism.

### Identifying the genetic overlap between the three major types of sperm defects

Focusing further on the spermatogenic defects indexed in male infertility, they were classified into three broad categories viz. sperm number defects, sperm motility defects and sperm morphological defects. A considerable genetic overlap was seen among these defects (Supplementary Figure S3).

Two hundred and twenty-nine genes were found to be associated with all three types of sperm defects. While the genes common to all three types of spermatogenic defects were enriched in cancer pathways, the genes unique to each of the three groups do not show any major pathways enrichment. The common genes are enriched in genes that play a role in biological processes involved in reproduction and development. The genetic overlap between genes associated with spermatogenic defects and cancer reiterates the risk of developing cancer in patients diagnosed with sperm defects.

### Disease-based analysis

The different validated male reproductive disease conditions included in MIK were broadly grouped into the following 14 groups. Citations where the disease condition was reported as male infertility were included in the male infertility (cause unknown) group. The disease groups excluding the male infertility (cause unknown) group with their top 10 associated genes, enriched pathways, comorbid conditions and enriched biological processes are given in Table 1.

**Table 1.** Disease-based analysis

Type of defects	Top 10 genes based on cited references	Enriched pathway (ToppGene)	Top 3 enriched diseases condition other than male infertility (ToppGene)	Enriched biological process (ToppGene)
1. Sperm number defects	AR, CFTR, FSHB, INHB, DAZ1, MTHFR, ESR1, FSHR, LHB, AMH	Pathways in cancer	i. Endometriosis ii. PCOS iii. Neoplasm of the endometrium	GO:0022414 Reproductive process
2. Sperm motility defects	IL6, TNF, CATSPER1, AR, SOD1, AKAP4, FSHB, GPX4, MTHFR	Pathways in cancer	i. Endometriosis ii. Thyroid cancer iii. Breast cancer	GO:0022414 Reproductive process
3. Sperm morphological defects	DPY19L2, SPATA16, CFTR, AR, AURKC, DNAH1, CFAP44, SPEF2, CFAP43	Pathways in cancer	i. Endometriosis ii. Leukemia iii. Neoplasm of the endometrium	GO:0022414 Reproductive process
4. Defects in vas deferens	CFTR, ADGRG2, EDNRA, SLC9A3, MCP, MMP-2, NOS, TGFB1, CACNA1S, PANK2	–	i. Lung diseases ii. Cystic Fibrosis	GO:0044703 Multi-organism reproductive process
5. Androgen insensitivity	AR, AMH, NCOA2, HBO1, FSHB, HSD17B3, LHB, SOX9, WT1	Regulation of Androgen receptor activity	i. Gonadoblastoma ii. Gonadal Dysgenesis iii. Sex Differentiation Disorders	GO:0046661 Male sex differentiation
6. Cryptorchidism	INSL3, AR, INSL3R, AMH, ESR1, FGFR1, WT1, CIRBP, PIWIL4, CYP1A2, CYP11B1	Pathways in cancer	i. Endometriosis ii. Endometrial Cancer iii. Malignant neoplasm of the endometrium	GO:0000003 Reproduction
7. Hypospadias	AR, SRD5A2, DGKK, NR5A1, ATF3, CYP11A1, MAMLD1, ESR2, AMH, GLI3	Nuclear Receptor transcription pathway	i. Endometriosis ii. Sex Differentiation Disorders iii. 46, XY Disorders of Sex Development	GO:0048608 Reproductive structure development
8. Hypogonadism	FGFR1, GNRHR, DAX1, KAL1, LHB, AMH, GNRH1, PROKR2, FSHB, INHB	Hormone ligand-binding receptors	i. Klinefelter Syndrome ii. Kallman syndrome	GO:0007548 Sex differentiation
9. Hypospermatogenesis	FSH, GAPDHS, INHB, PABPC3, PCNA, NDRG2, DFFRY, MAMLD1, CST8, SPAG5	Pathways in cancer	i. Nephroblastoma ii. Endometriosis iii. Multiple Myeloma	GO:0000003 Reproduction
10. Leydig cell dysfunction	LHR, CYP17A1, FSH, LHRH, IL6, AR, MEF2A, LHX9, SMURF1, TGFB1, TP53	Hormone ligand-binding receptors	i. Cancer ii. Female infertility	GO:0007548 Sex differentiation
11. Semen quality	AR, Fas, Eppin, SOD, CFTR, CGA, CHEK1, CLU	Pathways in cancer	i. Lung Neoplasms ii. Prostatic Neoplasms iii. Endometrial Carcinoma	GO:1901216 Positive regulation of neuron death
12. Testicular defects	INSL3, AR3, LGR8, SRY, TP53, ESR2, GATA4, AMH, INHB, SOX3	Nuclear Receptor transcription pathway	i. Klinefelter’s syndrome ii. Polycystic Ovary Syndrome iii. Endometrial Carcinoma	GO:0008584 Male gonad development
13. Varicocele	GSTT1, INHBA, FSH, AMH, HSPA4, HSP90AA1, FAS, BCL2, SOD1, CASP9	Pathways in cancer	i. Polycystic Ovary Syndrome ii. Ulcerative Colitis iii. Acute myocardial infarction	GO:0048608 Reproductive structure development
14. Sex Reversal, Sex development	NR5A1, SRY, AMH, HSD17B3, CYP17, GATA4, AR, ADCY2, TSARG7, CYP11A	Androgen biosynthesis	i. Disorders of Sex Development ii. Sex Differentiation Disorders iii. Gonadal Dysgenesis	GO:0007548 Sex differentiation

The disease-based analysis indicates shared genetic etiology between genes causing major male and female reproductive disorders. Enrichment of cancer pathways among these genes point out to high risk of cancers in people diagnosed with reproductive disorders. Agenesis of vas deferens is a coexisting condition in a small portion of cystic fibrosis patients. The genes associated with endometriosis in female share a genetic overlap with genes implicated for cryptorchidism in males. These genes are enriched in cancer pathways, thus suggesting an increased risk of cancer in patients with cryptorchidism. Hypospadias, hypogonadism, leydig cell function, testicular defects and sex reversal/sex differentiation were mostly attributed to disrupted pathways like hormone ligand-binding receptors, nuclear receptor transcription pathway and androgen biosynthesis pathway that play a role in the proper functioning of the different reproductive processes in human.

### Genetic overlap between male reproductive disorders in MIK and other major disease classes

**Figure 7.**
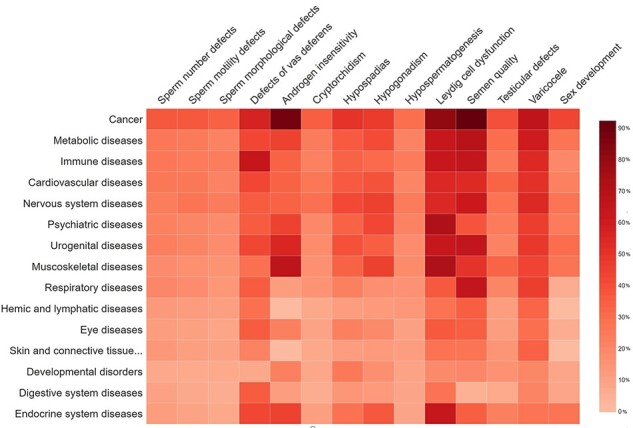
The percentage of gene overlap between disease subgroups in MIK and other disease classes.

The disease-based information in MIK was analyzed to understand the genetic overlap and common pathways between genes associated with male reproduction and other diseases. The disease grouping in MIK is obtained from the MeSH (29) database and the GAD database. The genetic overlap between different reproductive conditions causal for male infertility and the non-reproductive disease groups are represented as a heatmap in Figure 7. The genes present in MIK are pleiotropic in nature and show an overlap with diseases other than those of the reproductive system. Cancer is a common disease that shares high percentage of genetic overlap with all male infertility diseases, thus corroborating reports indicating high incidence of cancers in people with male infertility defects. Although few male infertility-associated genes like *AR, BRCA2, MSH5, MLH3,* and *LIG4* have been implicated in several cancers, the information is inadequate. An understanding of the shared mechanisms between male infertility diseases and cancers is imperative for predicting the risk of cancers and efficient clinical management in individuals suffering from male infertility (49). Additionally, the information in MIK also indicates a strong association between inflammatory diseases and defects in vas deferens, thus substantiating reports of an increased risk of inflammatory diseases and cancers (49, 50) in patients with complete or partial absence of vas deferens broadly termed as a congenital absence of the vas deferens (51).

Higher the percentage of the gene overlap the darker the shade of red.

Another interesting observation from disease–disease association data in MIK was the high percentage of genetic overlap between muscoskeletal diseases and male infertility diseases (leydig cell dysfunction and androgen insensitivity) in MIK. The percentage of causal genes associated with leydig cell dysfunction and androgen insensitivity in MIK is small as compared to the other male infertility phenotypes. Despite the small number, these genes showed an enrichment in muscoskeletal diseases. Although these genes have been independently cited in the literature to be associated with male infertility phenotypes and muscoskeletal diseases, the underlying causal mechanism and correlation between these diseases is unclear and need to be explored further.

An enrichment of pathways among genes common with male infertility (all genes in the validated dataset) and each of the top 15 associated disease conditions was carried out using ClueGO plugin in Cytoscape (Supplementary Figure S4a–d). While cancer pathways dominated among genes common to male infertility and cancer, the interleukin 4 and 13 pathways were the most enriched and central in all disease groups excluding developmental and digestive diseases. Interleukins are a class of proteins produced by many cell types, which function either in an autocrine and/or paracrine mode on binding to their cell surface receptors. The interleukin pathways were found to be central among all the associated conditions. The genes in this pathway play a role in regulating immune responses, cell proliferation, survival and translational control. Most associated diseases in MIK are homeogryphic in nature and are affected by the dysregulation of the genes in the interleukin pathways.

Arachidonic acid metabolism pathway, known to be important for sperm motility (52, 53), was enriched in obesity, diabetes, non-alcoholic fatty liver disease, cardiovascular and immune diseases (54) in MIK. Its role in linking nutrient metabolism to immune and inflammation pathways may be associated with the development of these diseases. SUMOylation is an enriched and shared pathway between cardiovascular, nervous system and male reproductive diseases. This pathway is important for post-translational modification of genes important for post-testicular sperm maturation and reproduction (55). It is also crucial for regulation of genes involved in cell survival and proliferation in the heart tissue (56). Several nervous system-associated proteins are modified by SUMOylation for its proper functioning and hence is a crucial process in a wide range of neurological and neurodegenerative diseases (57).

Steroid metabolism pathway is another common pathway shared between most of the associated diseases. While the genes in the steroid metabolism have been implicated in male infertility (58, 59), they are also involved in heart-specific steroid metabolism that plays a role in cardiac hypertrophy (60), in development of obesity and type 2 diabetes (61), and muscoskeletal and psychiatric disorders. Neurosteroids are known for their protective effect in neurodegenerative diseases, and their altered signaling has been associated with anxiety and depression (62). The gonadal steroid hormones are also implicated in development of age-related musculoskeletal disorders (63).

Psychiatric diseases also share genes associated with male infertility that play a role in the ERBB4 pathway. The interaction of members of both ERBB4 and the NRG families is associated with psychiatric diseases like schizophrenia (64).

The nuclear receptor transcription pathway was observed to be enriched among metabolic diseases. Genes in this pathway regulate adrenal and gonadal development, steroidogenesis and the reproductive axis (65). These receptors are also expressed in skeletal muscle and are important for glucose tolerance and improve metabolic conditions like insulin resistance and dyslipidemia (66).

Some of the other enriched pathways include the HSP90 cycle for steroid hormone receptors that regulates protein misfolding in case of nervous system diseases (67), insulin-like growth factor 1 receptor (IGF1R) signaling pathway affecting the rheumatoid arthritis pathogenesis (68) and the PI3K pathway that is crucial for embryonic development and is enriched in developmental disorders (69)

Defects in genes affecting the fertility of men have also been suggested to affect the general health of an individual. It is imperative to understand the shared genetic etiology and pathways common between the different male factor defects for prognosis and effective management of the patients. The disease and pathway information can be explored to understand the shared genetic etiology between male factor defects and other non-reproductive disorders.

### Case Study: predicting candidate genes for cryptorchidism using gene prioritization and a network-based approach

One hundred and forty-nine genes were identified as possibly associated with cryptorchidism with evidence from more than two sources. The genes are listed in Supplementary Table S1. The Human Protein Atlas database and the PubMed database were searched for tissue expression patterns of these genes and literature association with cryptorchidism, respectively. These data are represented as a Venn diagram (Supplementary Figure S5).

Twelve (*PIWIL1, BAX, CPEB1, HSD17B1, ATRX, DMRT1, ZBTB20, DAZL, EGFR, CHEK2, CALCA, NSMF*) of the 149 genes shortlisted based on evidence-based scoring had some direct or indirect reported association with cryptorchidism. Although *PIWIL1* has not been reported to be associated with cryptorchidism, the gene expression in members of the PIWI pathway in cryptorchidism has been reported (70). The expression of *Bax* in undescended testis (71), *EGFR* in leydig cells of cryptorchid boys (72), has been reported to be dysregulated when compared with controls. *CALCA* is one of the genes that play a role in the proper development and functioning of the gubernaculum and its dysregulation may lead to cryptorchidism (73). A *DAZL* polymorphism was identified in a patient with cryptorchidism and was presumed to be the causal genes for cryptorchidism in the reported case (74).

Few of the genes like *CPEB1, HSD17B1, NSMF, ATRX, DMRT1, CHEK2 and ZBTB20* have been associated either with syndromes having cryptorchidism as one of their phenotypic manifestations or are implicated in cryptorchidism-associated disorders. *CPEB1* at the 15q25.2 locus was associated with congenital diaphragmatic hernia (CDH) (75). *HSD17B1* and *DMRT1* although not directly implicated are reported to be associated with testicular germ cell tumor that has frequently been associated with cryptorchidism (76, 77). *NELF* has been associated with Kallmann syndrome (78), *ATRX* polymorphism has been associated with ATR-X syndrome (79), *CHEK2* is a candidate gene for male breast cancer (80) and *ZBTB20* variants has been reported to be the causal gene in five unrelated individuals with Primrose syndrome. In 78% of cases with Primrose syndrome, cryptorchidism is one of the manifestations (81).

The majority of the 149 novel predicted genes were found to be involved in regulatory processes (Supplementary Figure S6). The two major enriched pathways among the predicted novel genes are the androgen receptor signaling pathway and corticotropin-releasing hormone signaling pathway (Supplementary Figure S7). Development of the fetal gubernaculum is dependent on insulin-like three and androgen and is essential for testicular descent. Additionally, gubernacular defects were observed to impede muscle development, cytoskeletal function and androgen-regulated pathways in rats (82). Gene expression levels of members of the corticotropin-releasing hormone family have been observed to be altered in cryptorchidism (83). The predicted genes and enriched pathways need to be experimentally validated to verify their role in cryptorchidism and to gain a better understanding of the genetic etiology of cryptorchidism.

## Conclusion

The MIK is a catalogue of genes associated with the different reproductive conditions leading to male infertility. It holds information on 1564 validated and 16 190 predicted genes associated with male infertility. The knowledgebase is a comprehensive resource with genes annotated with disease association information manually curated and compiled from reference literature and functional information-like gene ontology, pathways and diseases mined from publically available databases. The knowledgebase provides access to tools for both gene and sequence-based analysis for genes present in MIK. The data in the knowledgebase would facilitate identification of novel candidate genes and pathways crucial for the different male reproductive disorders leading to infertility. In addition, it will also aid in delineating shared genetic etiology between male infertility phenotypes and other non-reproductive disorders. A subset of genes associated with cryptorchidism in MIK was used to identify novel genes and prioritize them for their association with cryptorchidism using gene prioritization and network analysis approach. The in silico analysis identified 149 novel candidate genes that could be probable candidate genes for cryptorchidism. Thus, this resource will facilitate in identification of novel candidate genes associated with male infertility, understanding the shared pathways between diseases and also help in delineating the high-risk conditions associated with male infertility.

## Supplementary Material

baab049_Supp
