# Supplementary figures and images for: Male Infertility Knowledgebase: decoding the genetic and disease landscape
Source: Database (Oxford). 2021 Aug 7;2021:baab049. doi: 10.1093/database/baab049 (PMC8346693; doi:10.1093/database/baab049)

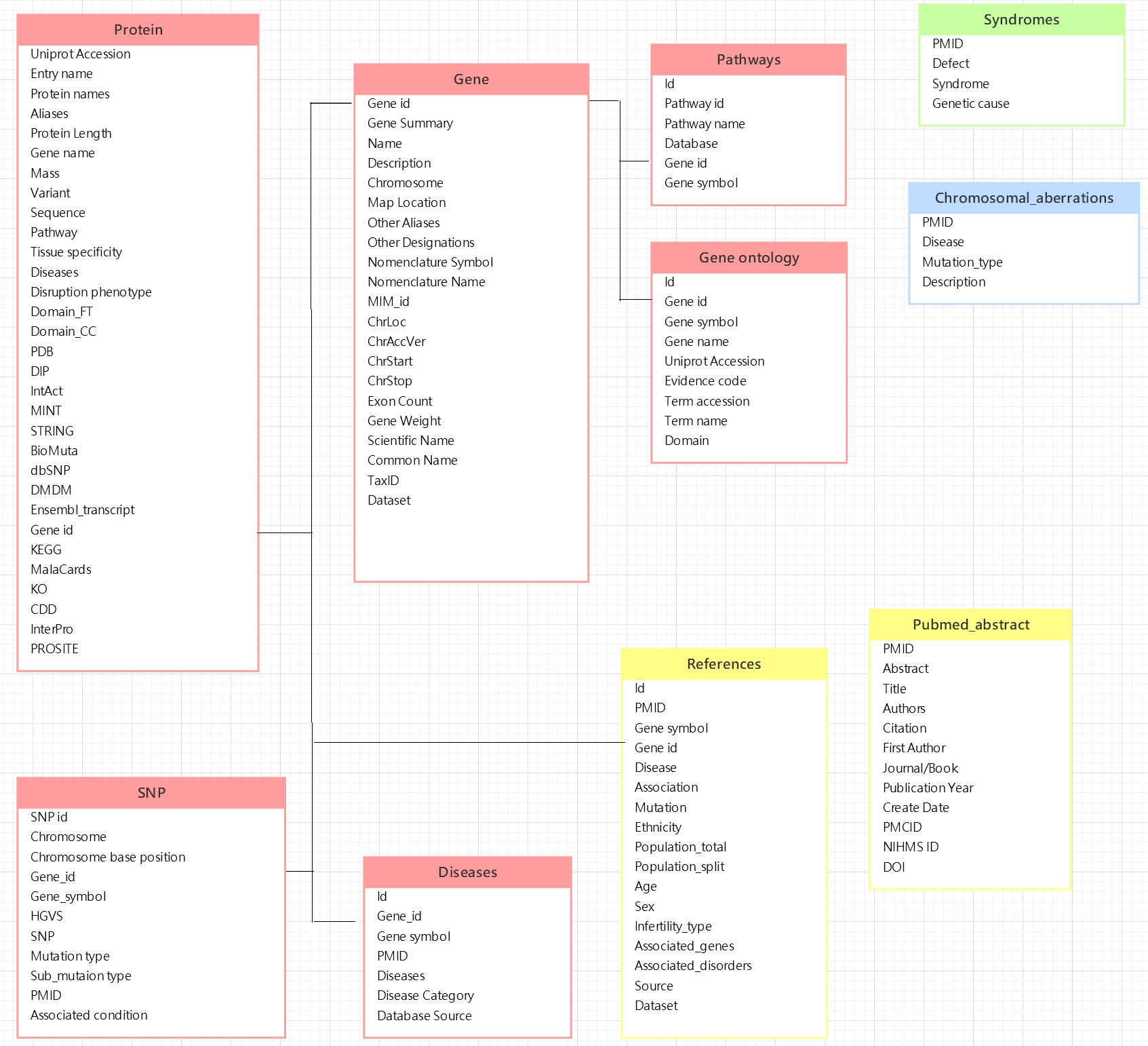

Supplement: baab049_Supp [file baab049_supp.zip › Supp_Fig2.jpg]

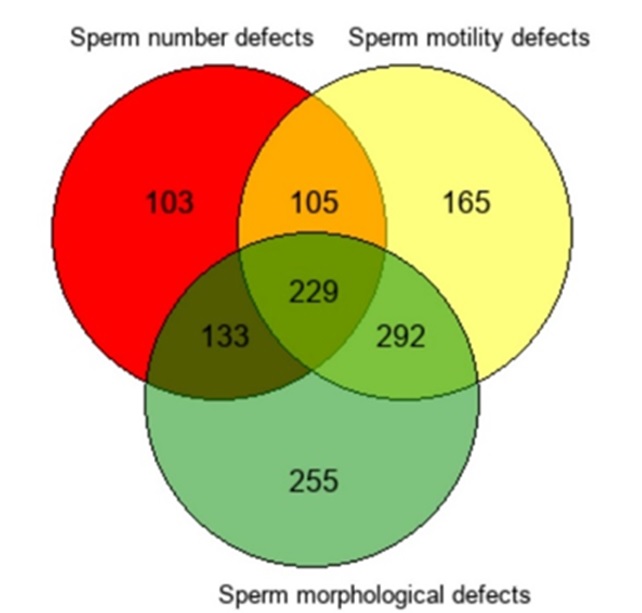

Supplement: baab049_Supp [file baab049_supp.zip › Supp3.jpg]

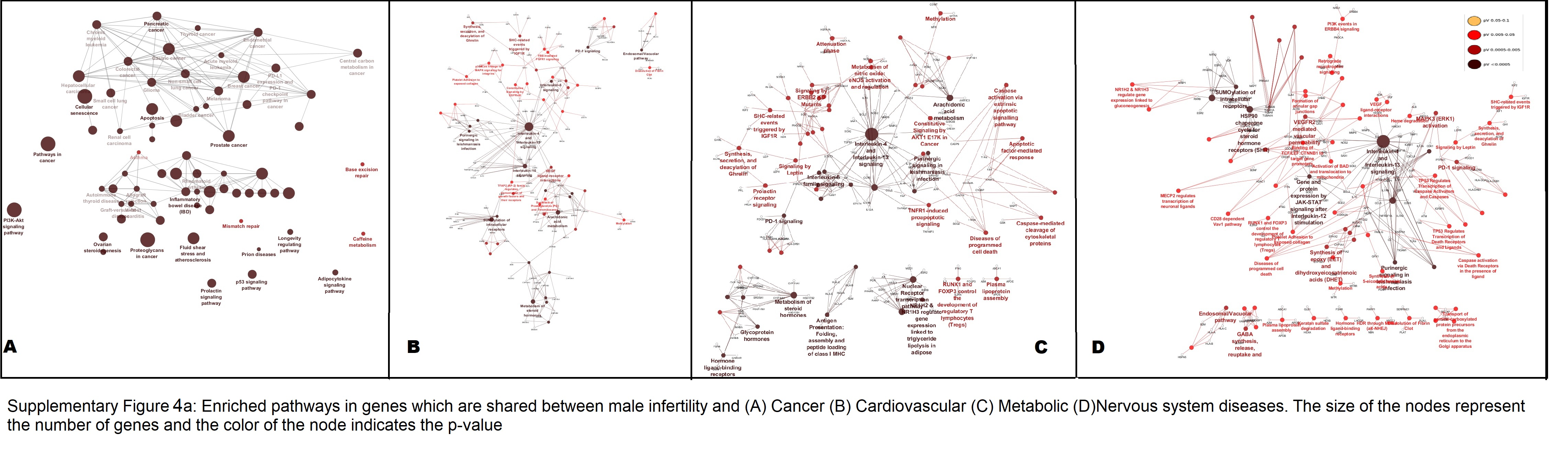

Supplement: baab049_Supp [file baab049_supp.zip › suppl4_a.tif]

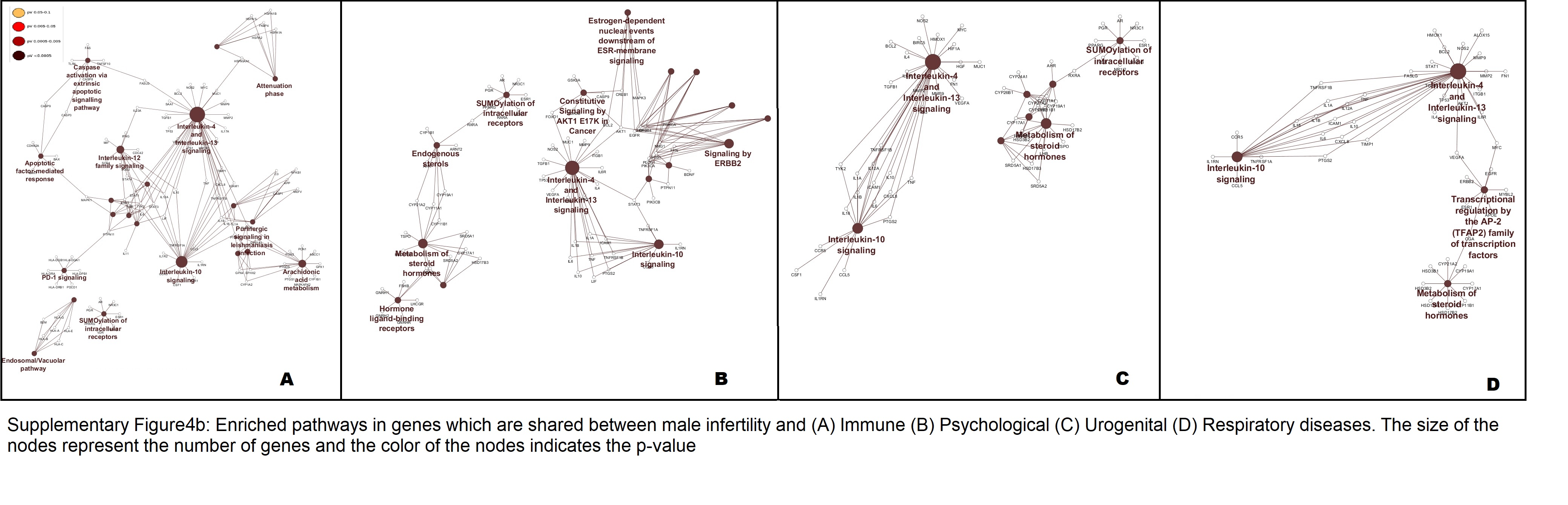

Supplement: baab049_Supp [file baab049_supp.zip › suppl4_b.tif]

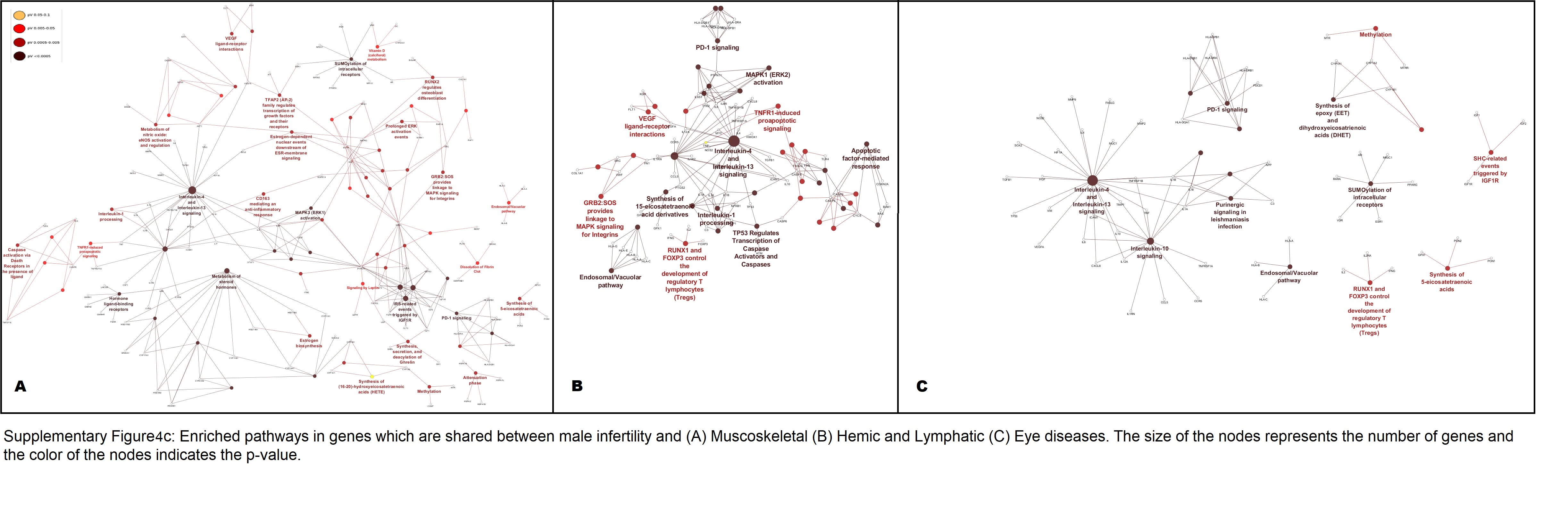

Supplement: baab049_Supp [file baab049_supp.zip › suppl4_c.tif]

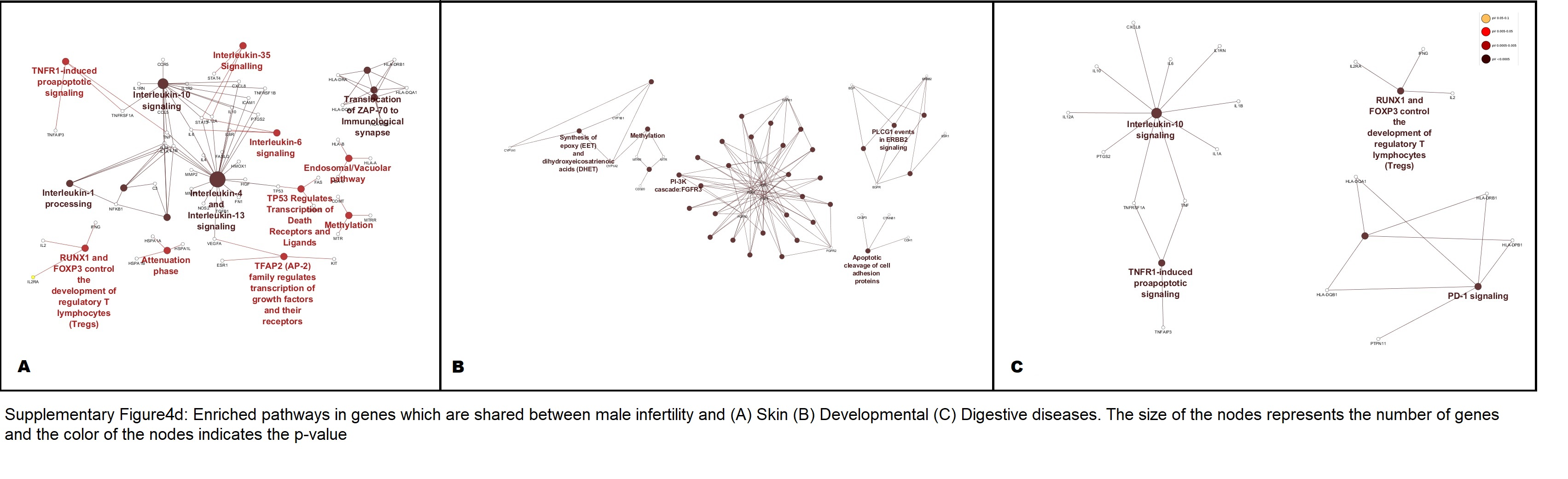

Supplement: baab049_Supp [file baab049_supp.zip › suppl4_d.tif]

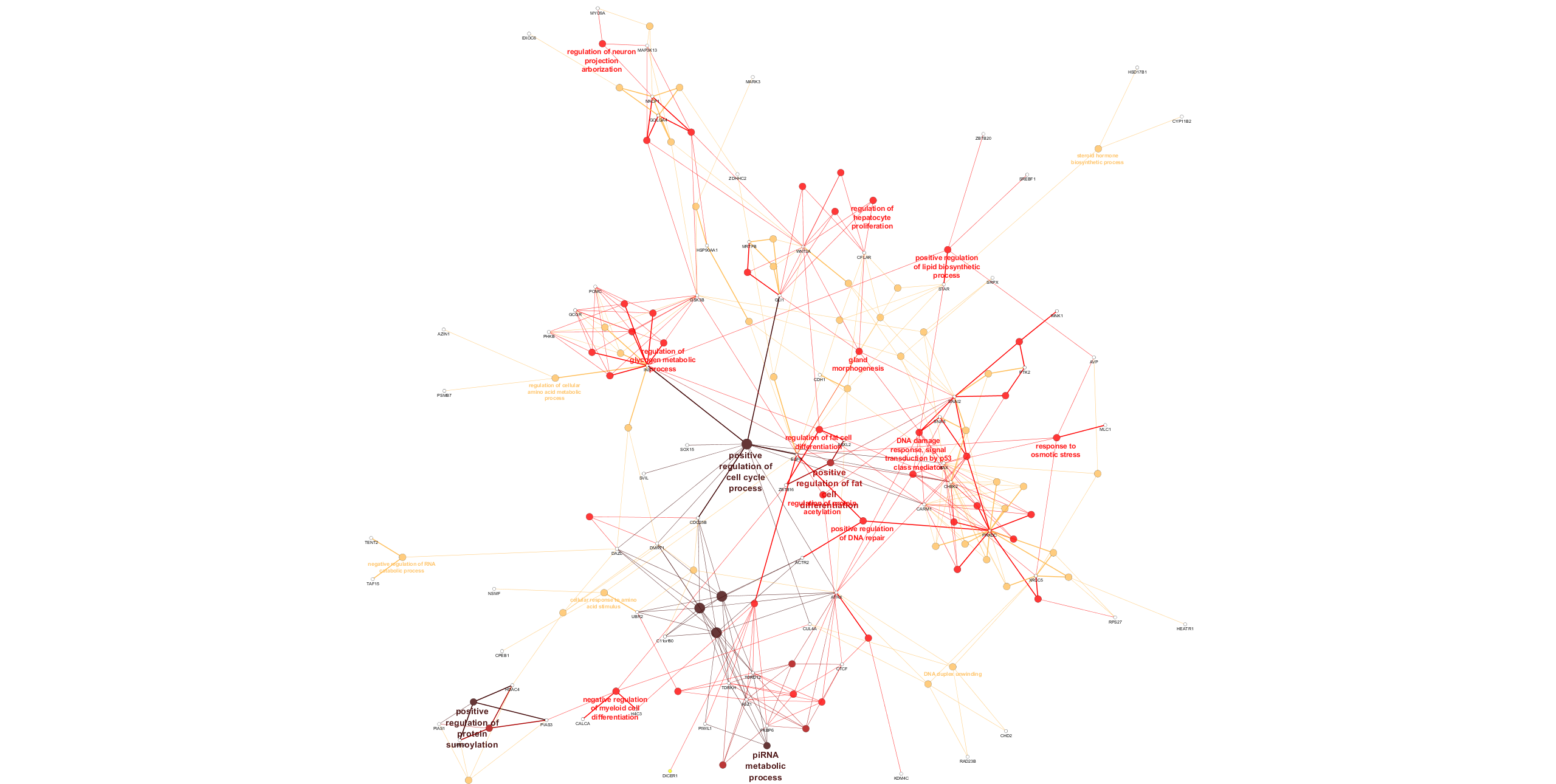

Supplement: baab049_Supp [file baab049_supp.zip › supplementary_6 (2).png]

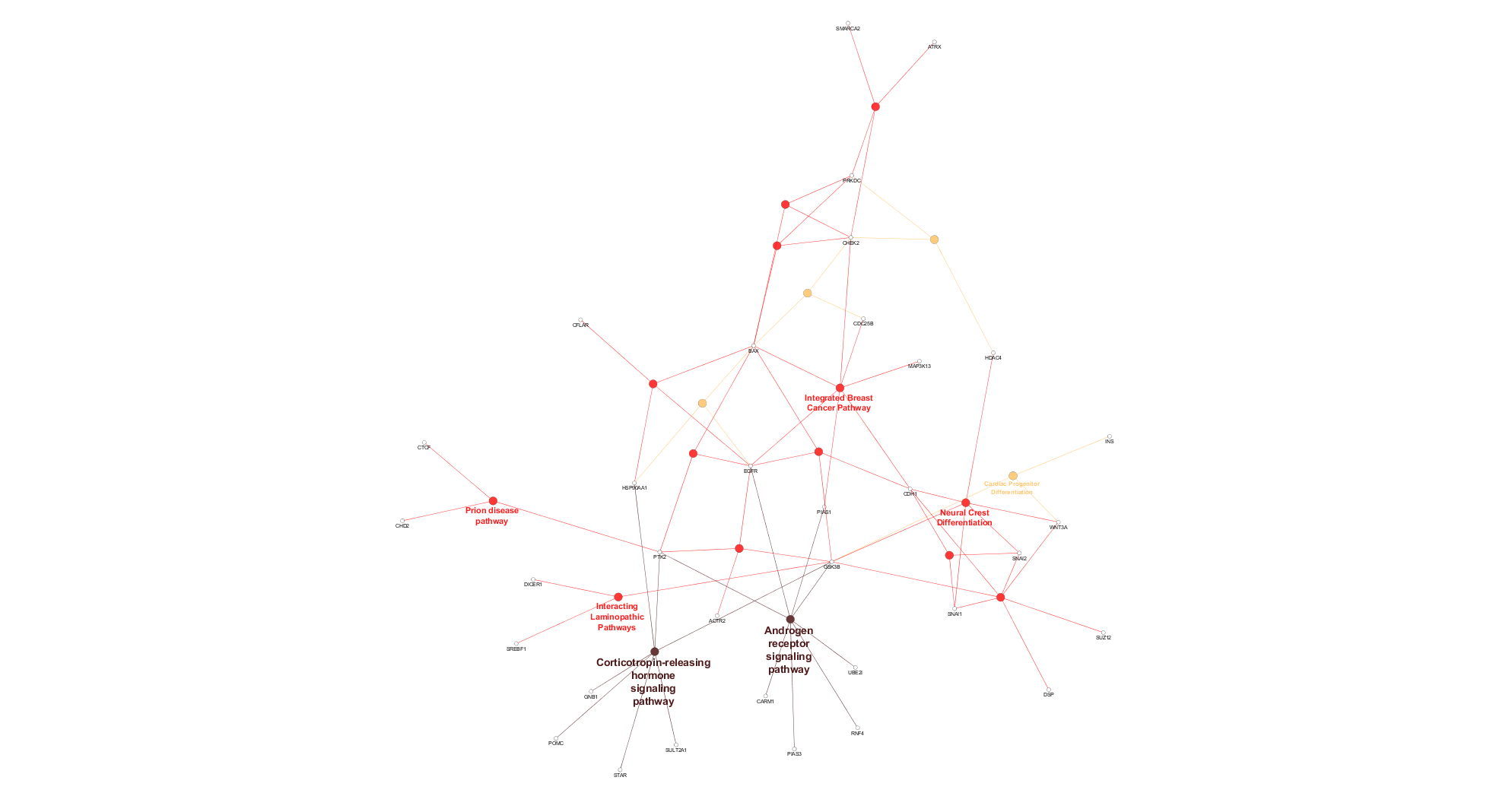

Supplement: baab049_Supp [file baab049_supp.zip › supplementary_7.png]

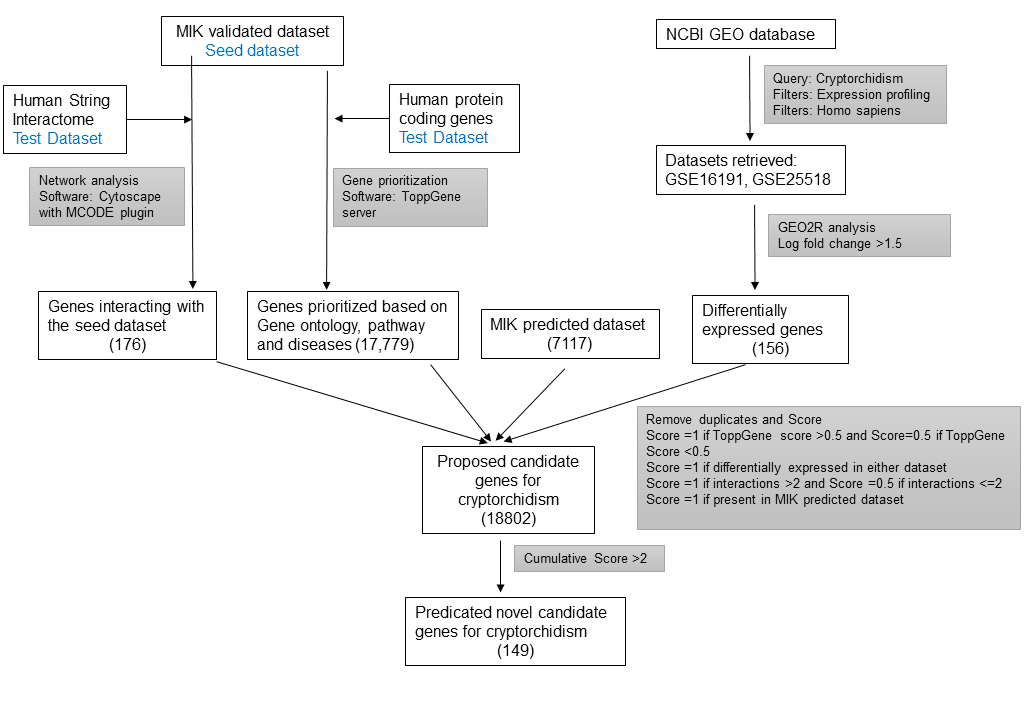

Supplement: baab049_Supp [file baab049_supp.zip › supplementary_figure1.tif]

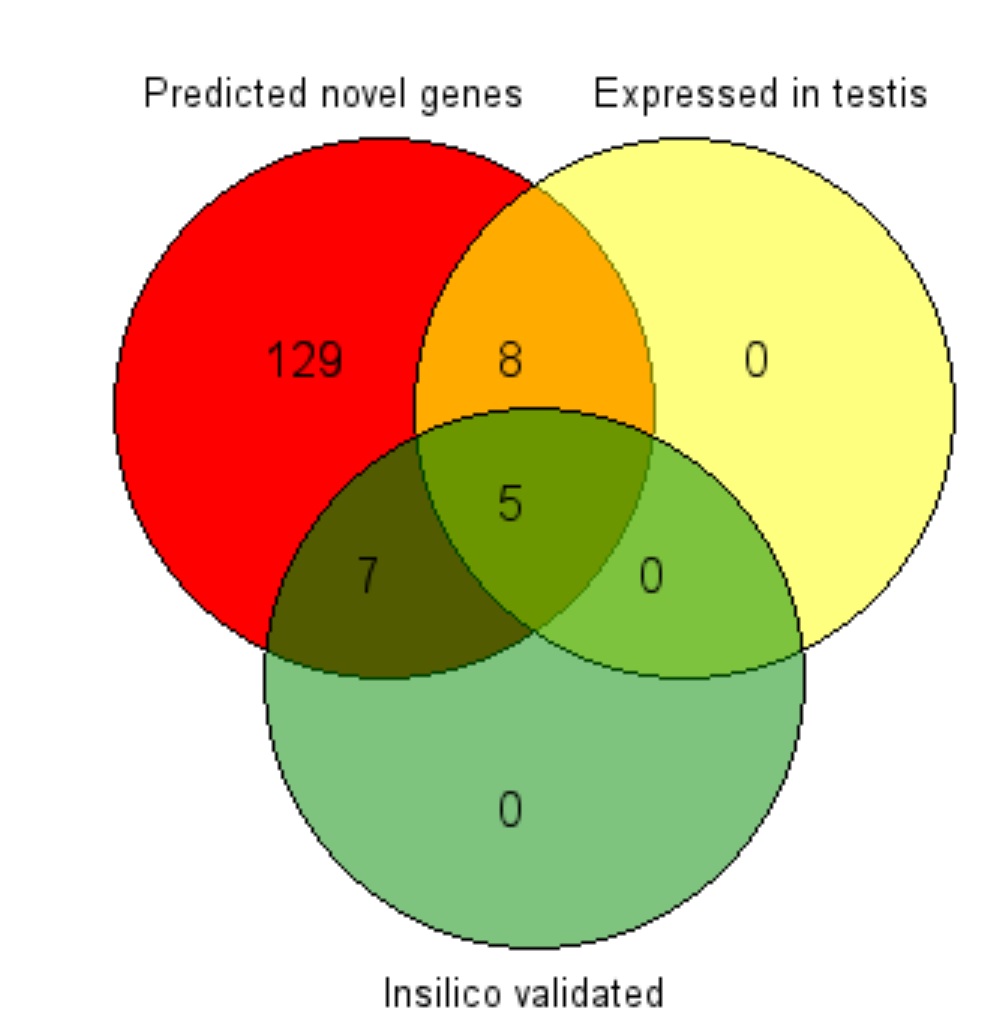

Supplement: baab049_Supp [file baab049_supp.zip › supplementaryfigure5.jpg]
